# Guided mindfulness meditation as a priming strategy for reducing anxiety and facilitating motor skill learning

**DOI:** 10.3389/fpsyg.2026.1697956

**Published:** 2026-04-16

**Authors:** Merwyn Yu Xian Lee, Megan Wan Ting Tay, Meng Leong Chia, Pei Ling Choo, Xiang Ren Tan

**Affiliations:** 1 National University Hospital, Singapore, Singapore; 2 Health and Social Sciences Cluster, Singapore Institute of Technology, Singapore, Singapore; 3 Ng Teng Fong General Hospital, Singapore, Singapore; 4 Tan Tock Seng Hospital, Singapore, Singapore; 5 Heat Resilience and Performance Centre, Yong Loo Lin School of Medicine, National University of Singapore, Singapore, Singapore; 6 Human Potential Translational Research Programme, Yong Loo Lin School of Medicine, National University of Singapore, Singapore, Singapore

**Keywords:** attention, cognitive function, motor learning, movement, state anxiety

## Abstract

**Introduction:**

Mindfulness meditation (MM) is a promising cognitive strategy that may facilitate motor learning by improving attention and reducing anxiety. Our study aimed to investigate the effects of guided MM on motor learning in healthy young adults.

**Methods:**

The study was conducted as randomised controlled trials where 24 meditation-naive participants underwent either a 10-min MM session or seated rest (CT) before performing circle tracing tasks using their (1) trained (dominant) hand, (2) untrained (non-dominant) hand, and (3) untrained hand to trace with an inverted screen display (a novel motor task). Post-learning, they performed a Parametric Go/No-Go (PGNG) cognitive test. Anxiety level was assessed via heart rates, State–Trait Anxiety Inventory – State subscale and Visual Analogue Scale-Anxiety (VAS-A).

**Results:**

Heart rates were lowered during guided MM (*F*_3.3,36.6_ = 3.495, *p* = 0.022) with reduction in VAS-A scores (*p* = 0.012) after a single session of MM, indicating lowered anxiety. Only the MM group demonstrated learning effects for the trained hand task (+4.2% in accuracy; *p* < 0.001) while no differences were observed for the untrained hand and novel motor learning tasks. PGNG test results revealed that cognitive functions such as inhibitory control, sustained attention, and processing speeds remained unaltered between groups.

**Discussion:**

Our findings suggest that a brief guided MM session reduced anxiety levels and facilitated the learning of simple motor tasks. Future studies may explore the use of extended MM sessions and/or regular meditation practice to elicit cognitive benefits for facilitating novel or complex motor learning.

## 1 Introduction

Motor skill learning involves the learning of specific muscle movements for performing a task and is indispensable for developing fundamental movement across lifespan, such as walking, grasping a pen, or holding a cup. Likewise, it is vital for the development of motor skill competence, which improve sports performance, enhance participation in more varied and fitness-oriented activities, and to maintain physical fitness in later years (Stodden et al., 2008; Robinson et al., 2015). It also supports the acquisition of new motor skills while in pursuit of task training, occupations or recreational activities (Voelcker-Rehage, 2008). Furthermore, motor skill relearning is critical for stroke survivors or spinal cord injury patients to promote functional movement recovery (Kapadia et al., 2020). However, the learning process requires sustained focus and concentration over extended periods, which can be especially challenging for learners with motor limitations, dyspraxia or poor attentional control.

According to the Fitts and Posner (1967) model, the process of motor skill learning consists of the cognitive, associative and autonomous stage. In the cognitive stage, movements are slow and inconsistent, requiring high levels of attentional demands (Weaver, 2015). Importantly, the attentional control theory (Eysenck et al., 2007; Derakshan and Eysenck, 2009) proposes that anxiety and worry drain attentional resources, lowering the processing efficiency in demanding cognitive tasks. Anxiety, as defined by the American Psychological Association, is an emotion characterized by feelings of tension, worried thoughts, and physical changes such as elevated blood pressure. Importantly, increased state anxiety (a transitory state) triggers conscious control of movements, destabilizes motor skills and disrupts attention by weakening goal-directed control and increasing stimulus-driven processing (Mullen and Hardy, 2000; Eysenck et al., 2007; Wilson et al., 2009). Therefore, learning or performance anxiety can degrade one’s concentration and impede motor skill learning.

Motor skill learning involves observation, precise action selection and controlled motor execution underpinned by effective attentional control (Song, 2019). Therefore, this may be facilitated by a cognitive strategy such as mindfulness meditation (MM) which is characterised by bringing cognitive processes under voluntary control by adopting a non-judgemental and calm attitude, acknowledging and accepting one’s feelings, thoughts, and bodily sensations, and focusing one’s awareness on the present moment (Brown and Ryan, 2003; Kabat-Zinn, 2003). MM has been associated with positive effects on learning and memory processes, cognition, emotion control, and self-awareness of an individual (Vago and David, 2012; Roemer et al., 2015; Lueke and Lueke, 2019; Gawande et al., 2023; Zainal and Newman, 2024). Brief MM can improve emotional processing including reduction of emotional intensity and emotional attention bias and aid in lowering of apprehension and anxiety (Call et al., 2014; Wu et al., 2019). Thus, MM practice may benefit learners during the cognitive stage of motor learning where a calmer state of mind facilitates a longer period of focus.

MM enhances components of attention like sustained vigilance and executive control (Semple, 2010; Zhong et al., 2024). It has been theorised in the Liverpool Mindfulness Model (Malinowski, 2013) that MM practice hones the mental core processes encompassing attention, emotional flexibility and cognitive flexibility. Earlier studies have reported that focused attention meditation, characterised by maintaining attention on a specific instructed object (e.g., breath or body awareness), improves cognitive control during motor sequence performance (Chan et al., 2018; Chan et al., 2020). An electroencephalography study (Moore et al., 2012) showed that regular brief MM training for 16 weeks led to improvements in the focusing of attentional resources during Stroop task. Likewise, a single MM session can benefit attentional control where (Gao and Zhang, 2023) showed that performing 20-min MM before competition improved task-relevant gaze behaviours in college athletes, translating to better shooting performance. These studies demonstrate an improvement in the regulation and focusing of attention after brief MM practice. However, it remains unclear whether this attentional benefit brought about by MM can facilitate motor skill learning.

While there has been research on the effects of meditation to augment motor learning and performance, it is still unclear if the conduct of a brief MM session preceding the task is able to facilitate motor learning. It has been demonstrated that even a 10-min session of MM was sufficient to improve the allocation of attentional resources in novice meditators (Norris et al., 2018). Notably, this highlights the potential of a brief MM session as a priming strategy to enhance motor learning through the reduction in anxiety and improvement of attention. Therefore, our study aims to investigate the effect of a single session of guided MM on motor learning in healthy young individuals. We hypothesised that a brief MM session before motor learning helps to lower state anxiety level and improve the motor learning performance as compared to control seated rest. We further postulate that a lower anxiety state benefits various cognitive functions including inhibitory control, sustained attention and processing speeds, which facilitate motor learning. If proven effective, MM may be used as a priming strategy for athletes to enhance motor skills learning, or as an adjunct rehabilitation strategy in clinical populations.

## 2 Materials and methods

### 2.1 Participants

Healthy young adults aged between 18 and 35 years old who can read, write and effectively communicate in English were recruited in this study. Participants were excluded if they were ambidextrous, involved in any meditation-related practices such as MM, transcendental meditation, insight meditation, Vipassana meditation, Zen meditation, Qigong, Tai Chi or Yoga in the past 6 months, had history of neurological illnesses, psychological disorders or learning disability or currently taking any psychoactive substances, alcohol or using tobacco. Prior to the commencement of the study, participants attended an online informed consent session where the study details were explained before they provided their written consent. This study was approved by the Singapore Institute of Technology Institutional Review Board (SIT-IRB, Ref no. 2021109) and the conduct of this study adhered to the ethical principles outlined in the Declaration of Helsinki. Written informed consent was obtained from all participants.

### 2.2 Procedure

**Figure 1:**
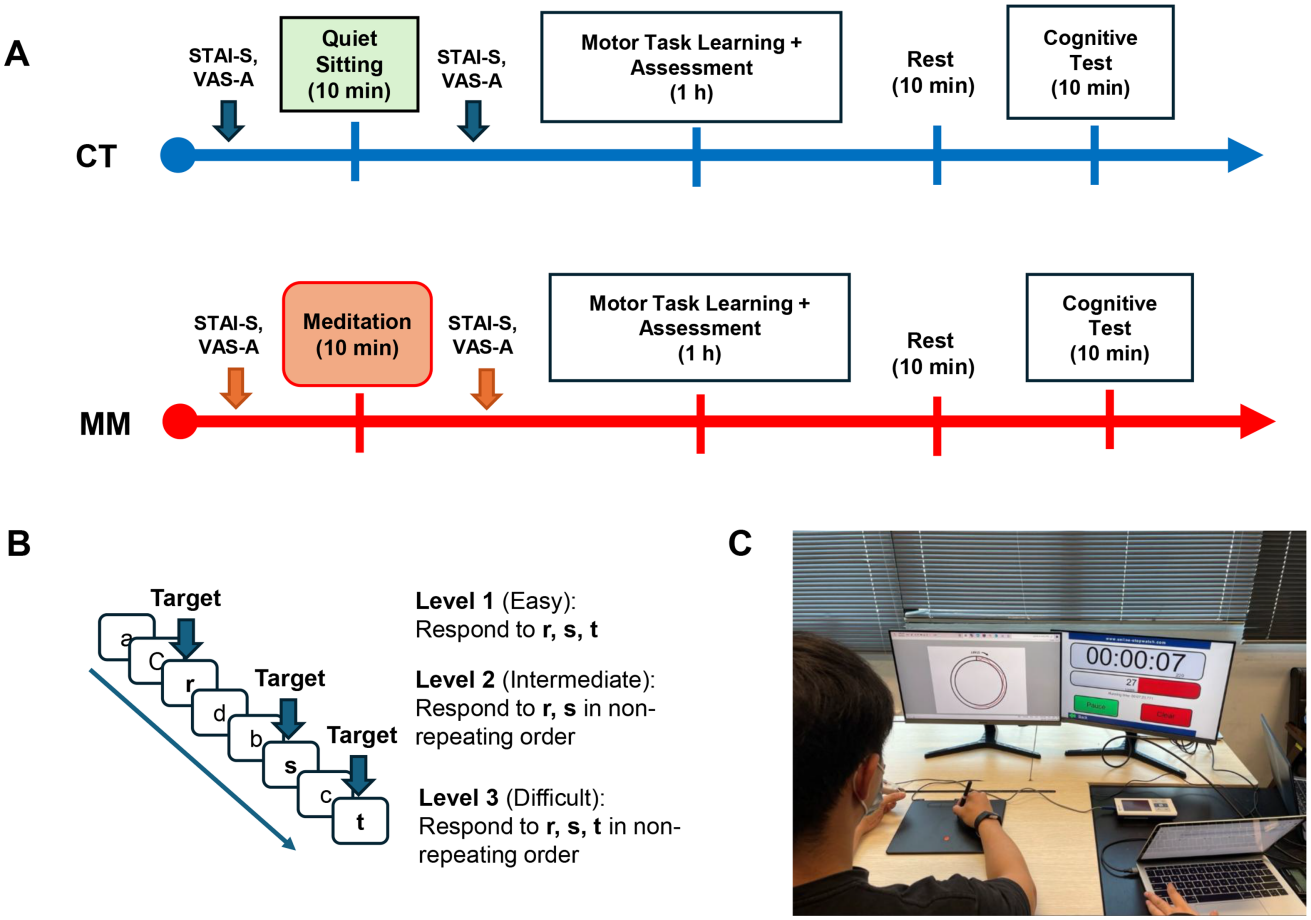
**(A)** Experimental design for CT (control) and MM (mindfulness meditation) groups. **(B)** Example test stimuli for parametric Go/No-Go test with graded levels of difficulty. **(C)** Motor learning task (circle-tracing) with digitizer and monitor screen. STAI-S, State–Trait Anxiety Inventory – state subscale; VAS-A, visual analogue scale-anxiety.

The study was conducted as randomised controlled trials (Figure 1A) where participants were randomly assigned by the research team to either the intervention (MM) or control (CT) group, using an online random generator tool.^1^ For the MM group, the participants underwent a 10-min session of guided MM through an audio recording freely available from the mobile application – *Headspace*, a recognised meditation application. The selected recording was an introductory module on the basics of MM, including body scan techniques and simple breathing exercises.^2^ Participants were provided with the same headphones to use, with audio volume standardised at 70% to ensure consistency of intervention. For the CT group, the participants underwent a 10-min session of quiet sitting instead and were instructed not to use their phones or fall asleep. The implementation of a passive control in our study was intended to represent a typical situation wherein participants do not engage in any active strategy prior to a motor learning task. Both the MM and CT sessions were monitored non-obtrusively by the investigator seated at a distance away from the participants. Participants were asked to rate on their anxiety level on the Visual Analogue Scale-Anxiety (VAS-A) tool and the State–Trait Anxiety Inventory – State subscale (STAI-S) questionnaire before and after the guided MM/quiet sitting session. Their heart rates (HR) were monitored with a pulse oximeter throughout the entire study.

All participants were given a 5-min period to familiarise themselves with the environment before the commencement of the experiment. Participants then underwent 10 min of guided MM or quiet sitting before they proceeded to perform a series of motor learning tasks. After completing the tasks, they were given a 10-min rest period. Thereafter, they performed a cognitive test - Parametric Go/No-Go (PGNG) test on a computer via a software platform (Inquisit 5 Lab, Millisecond, Seattle, Washington).

### 2.3 Cognitive test

The PGNG test was used to assess their inhibitory control, sustained attention, and processing speeds. These cognitive functions are critical to motor learning processes where effective motor control relies on timely inhibition of motor impulsivity to ensure appropriate movements, while sustained attention allows a learner to maintain focus on the motor sequence and monitor their motor performance over time. Processing speed is vital as it allows the learner to identify erroneous actions promptly and perform required corrective motor behaviour during the learning process.

There are 3 levels in PGNG test (Figure 1B), designed with increasing difficulty where the last 2 levels have increasing inhibitory control demands. Level 1 (“Easy”) requires participants to respond to target alphabets “r,” “s,” “t” continuously regardless of order. This stage evaluates sustained attention and processing speed. Level 2 (“Intermediate”) requires participants to respond to target alphabets “r” and “s” in alternating or non-repeating order (i.e., if “r” was the target selected, the participant should only select “s” next and inhibit responses to “r” and “t”). Level 3 (“Difficult”) is similar to level 2, but adds on an additional target to detect, “t” (i.e., if “r” was the target selected, the participant should select either “s” or “t” next and inhibit response to “r,” vice versa). This level reduces the ability to anticipate the next appropriate response. The outcome measures of the PGNG test include (1) target detection (% accuracy for correct target trials), which relates to sustained attention, (2) reaction times, which relates to processing speeds, and (3) target suppression (% accuracy for correct inhibit trials), which relates to inhibitory control.

### 2.4 Motor learning tasks

To introduce appropriate motor learning tasks for healthy individuals, participants performed 3 different motor learning task blocks, which involved the tracing of circles within a set defined path (MovAlyzeR, NeuroScript LLC, United States) using a stylus pen on a digitizer board. A similar motor learning task has been employed in a previous study (Yadav and Mutha, 2016) demonstrating how deep breathing practice facilitates retention of newly learned motor skills in young healthy adults. The protocol was adapted and modified to create graded levels of difficulty, introducing both the unfamiliarity of using the non-dominant hand, and a novel learning aspect (i.e., tracing with an inverted display creates unfamiliar visuomotor dissonance). During the motor learning task blocks, participants were encouraged to complete the full tracing within the stipulated time as accurate as possible. The tracings were displayed in real-time on a computer screen (Figure 1C).

In the first motor learning task block (trained hand motor task), the participants performed 20 trials of circle tracing within the predetermined circular path with their dominant hand, where hand dominance was determined using Edinburgh Handedness Inventory – Short Form (Veale, 2014). Each circle tracing was timed using a stopwatch and the time taken for each tracing was used to determine the time limit for subsequent task blocks. In the second motor learning task block (untrained hand motor task), the participants performed 30 trials of circle tracing with their non-dominant hand. This was a time-restricted block where participants completed each tracing within the specific timeframe determined from their first task block. In the third motor learning task block (novel motor task), participants performed 30 trials of circle tracing with their non-dominant hand within an allocated time, but the screen monitor was inverted to introduce a novel learning stimulus. With the increased difficulty, participants were given twice the duration determined from the first task block to complete each tracing. The appropriate duration required to complete the circle tracing was determined through iterative pilot testing of the motor learning task blocks by the researchers prior to implementation. No training feedback (i.e., verbal comments from researchers on their performance or remaining time for each tracing attempt) was given to participants during the motor learning task blocks.

### 2.5 Motor performance and learning effect

Each tracing’s performance in the trained hand motor task was measured by the percentage of tracing that had landed within the defined path, expressed as % accuracy of circle tracing. Each tracing’s performance in the untrained hand and novel motor tasks was measured using a motor performance index (MPI). The creation of MPI by the authors was based upon a nuanced understanding of the specific test parameters, performance indicators, and the speed-accuracy trade-off effect in the tracing task (Heitz, 2014). MPI accounted for both the % accuracy of circle tracing and the % time taken to complete circle tracing within the stipulated time (%) expressed in the following formula:


$$\mathrm{MPI}=\frac{\%\text{accuracy of circle tracing}}{\%\text{time taken to complete circle tracing within stipulated time}}$$

The allocated tracing time for each participant represents the maximum limit for the % time taken (100%)—participants were stopped once this time was reached. Any impact on performance was therefore observed in accuracy, specifically if tasks were unfinished. Sensitivity analyses on MPI were conducted (Supplementary Table S1) and the changes in MPI scores differ minimally (within ±1%) alongside any percentage change in the individual parameters, reflecting the robustness of MPI as a performance indicator. In addition, no scaling effect was observed relating to percentage improvement across different allocated tracing times (Supplementary Table S2).

MPI > 1 indicates that the participant performed better than the allocated timing while MPI < 1 indicates that the participant performed poorer than the allocated time. This index was designed to account for adaptive changes on speed-accuracy trade-offs made by participants to complete the task. For instance, a lower MPI value will be computed in the scenarios where the participant (1) slowed down considerably to ensure accurate tracing, or (2) kept within the stipulated time but compromised on the accuracy of tracing. The short-term learning effect, defined as an improvement in motor performance, in each motor learning task block was assessed based on the difference between the mean data of the first 5 tracings (Pre) and the mean data of the last 5 tracings (Post).

### 2.6 Anxiety level

Anxiety leads to sympathetic activation that increases HR and hence, the elevation of HR constitutes one of the physiological indicators of anxiety (Hoehn-Saric and McLeod, 2000; Hoehn-Saric et al., 2004; Ahmadi et al., 2021). Therefore, the participants’ HR were monitored using a Nellcor handheld pulse oximeter throughout the study. HR values were recorded before and after each experimental procedure (i.e., guided MM/quiet sitting, motor learning tasks, cognitive test), at 1-min intervals throughout the guided MM/quiet sitting session, and at 5-min intervals throughout the motor learning and cognitive blocks. Data were collected at a higher resolution throughout the intervention period (brief MM) to enable detailed monitoring of HR changes. Each motor learning and cognitive task lasted approximately 10 min; HR measurements were taken at both the midpoint and upon task completion to ensure minimal disruption to participants’ engagement in task.

Psychological data on anxiety was assessed using the VAS-A tool (Luyk et al., 1988) and the STAI-S questionnaire - a subset of STAI (Spielberger et al., 1983). The VAS-A tool consists of a 10 cm continuous horizontal line on which participants were asked to rate their current level of anxiety, ranging from 0 (not anxious at all) to 10 (extremely anxious) by marking a cross on the line. The distance between the left starting point to the marked cross was used as a measure of the reported anxiety. Participants were asked to rate on the VAS-A scale before and after guided MM/quiet sitting. This tool has been validated as a quick way to measure state anxiety where it shows a moderate correlation with STAI (*r* = 0.56–0.59) and moderate-to-good reproducibility (ICC = 0.57–0.59) (Facco et al., 2013; Labaste et al., 2019).

STAI-S is a self-evaluation questionnaire consisting of a set of 20 questions that measures state anxiety (transitory state that fluctuates over time) anxiety by using items that measure subjective feelings of apprehension, tension, nervousness, worry, that are associated with arousal of the autonomic nervous system. The STAI-S score has a minimum of 20 points and a maximum of 80 points, where a higher score would indicate greater anxiety. Participants were asked to complete the STAI-S questionnaire before and after guided MM/quiet sitting. The STAI tool has good test–retest reliability (*r* = 0.70–0.88) and excellent internal consistency (*α* = 0.89–0.91) (Barnes et al., 2002). Using the collective data from three separate measures (HR, VAS-A and STAI-S), this allowed us to compare and cross-validate for the assessment of anxiety.

### 2.7 Statistical analysis

Estimation of required sample size was calculated with G*Power application (G*Power 3.1.9.7, gpower.hhu.de). Sample size was determined *a priori* based upon a previous study where participants showed improvement in motor performance (dart throwing; $\eta_{p}^{2}$ = 0.26) after training with mindfulness when compared with control (Zhang et al., 2016). This study was powered primarily for motor performance outcomes (primary outcome of interest) rather than cognitive measures. Power analysis showed that 24 participants was sufficient to provide 0.9 power for repeated measures analysis of variance (ANOVA; 2 participant groups, 3 timepoints) with α of 0.05 for significance.

Statistical Package for Social Sciences version 27.0 (SPSS Inc., Chicago, IL, USA) was used to analyse all data. Shapiro–Wilk test and Levene’s test were used to assess data normality and homogeneity of variance, respectively. Chi square analysis was performed to assess sociodemographic differences between groups. Repeated measures ANOVA was used to compare between groups on their HR profiles during intervention, motor learning tasks and cognitive task. Mauchly’s test was used to assess data sphericity, and the Greenhouse–Geisser correction was applied as necessary. Where significant interaction effects were established, pairwise differences were identified using the Bonferroni post-hoc analysis procedure adjusted for multiple comparisons.

Motor task performance (% accuracy and tracing time), MPI scores and cognitive task performance (% accuracy and reaction time) were analysed using linear mixed-effects models to account for repeated measurements within participants. Task performance or MPI score served as the dependent variable. Timepoint, task, group, and their interactions, where relevant, were included as fixed effects to test for condition-specific and group-level differences over time. Participant ID was modelled as a random intercept to capture individual differences in baseline MPI levels. The model was fitted using restricted maximum likelihood estimation. Model assumptions were evaluated using residual diagnostics, and fixed-effect estimates are reported with 95% confidence intervals. Paired *t*-test was used to compare trained hand task accuracy between pre- and post-intervention while Student’s *t*-test was used to compare changes in motor task performance between CT and MM. For STAI-S and VAS-A data, Mann–Whitney U test was used to compare between groups and Wilcoxon signed-rank test was used to compare between pre- and post-intervention within each group. For PGNG test, one participant’s target detection data was omitted due to erroneous performance. Significance was set at *p* < 0.05. Data is presented as mean (*M*) and SD for normal data, or median with interquartile range for non-normal data.

## 3 Results

### 3.1 Demographics

Twenty-four participants (*M* = 23.3 years old, *SD* = 2.2) were randomly assigned to the MM (*n =* 12; 7 males, 5 females) or CT (*n =* 12; 3 males, 9 females) group. There were no differences observed in the sex distribution [*Χ^2^*(1, *n =* 24) = 2.74, *p* = 0.098] and hand dominance [*Χ^2^*(1, *n =* 24) = 0.38, *p* = 0.537] between both groups.

### 3.2 Heart rate

**Figure 2:**
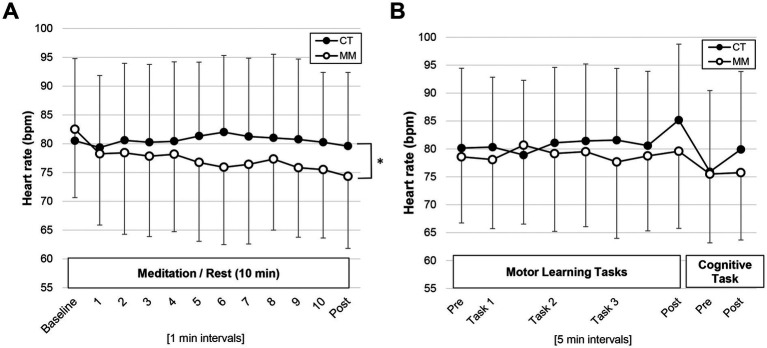
Heart rate profiles monitored during the **(A)** mindfulness meditation or control rest (quiet sitting) session, the **(B)** motor learning tasks and the cognitive task. CT, Control; MM, Mindfulness meditation. *Denotes group-time effect *p* < 0.05.

HR was lowered (by ~5 bpm) during meditation in MM as compared to CT group during quiet sitting where significant group-time interaction effect was observed (*F*_3.3,36.6_ = 3.495, *p* = 0.022, $\eta_{p}^{2}$ = 0.241; Figure 2A) with a growing reduction in HR across time. However, there was no difference in mean HR between groups before the commencement of the motor learning tasks (CT: *M* = 80 bpm, *SD* = 13; MM: *M* = 79 bpm, *SD* = 13; *p* = 0.771). HR profiles were comparable between groups during the motor learning and cognitive task phases (*p* > 0.05; Figure 2B).

### 3.3 VAS-A and STAI-S

In the CT group, VAS-A scores remained similar after quiet sitting [Pre: 0.08 (0.02–0.32); Post: 0.06 (0.00–0.06); *p* = 0.484]. However, in the MM group, there was a reduction in VAS-A scores [Pre: 0.38 (0.00–0.93); Post: 0.00 (0.00–0.22); *p* = 0.012] after a single session of guided MM. In the CT group, the STAI-S scores remained similar after quiet sitting [Pre: 25.50 (23.00–34.50); Post: 28.00 (22.00–32.50); *p* = 0.593]. In the MM group, there was also no difference observed in the STAI-S scores after a single session of guided MM [Pre: 24.50 (23.00–28.50); Post: 21.0 (20.00–28.50); *p* = 0.170].

### 3.4 Motor learning performance

**Figure 3:**
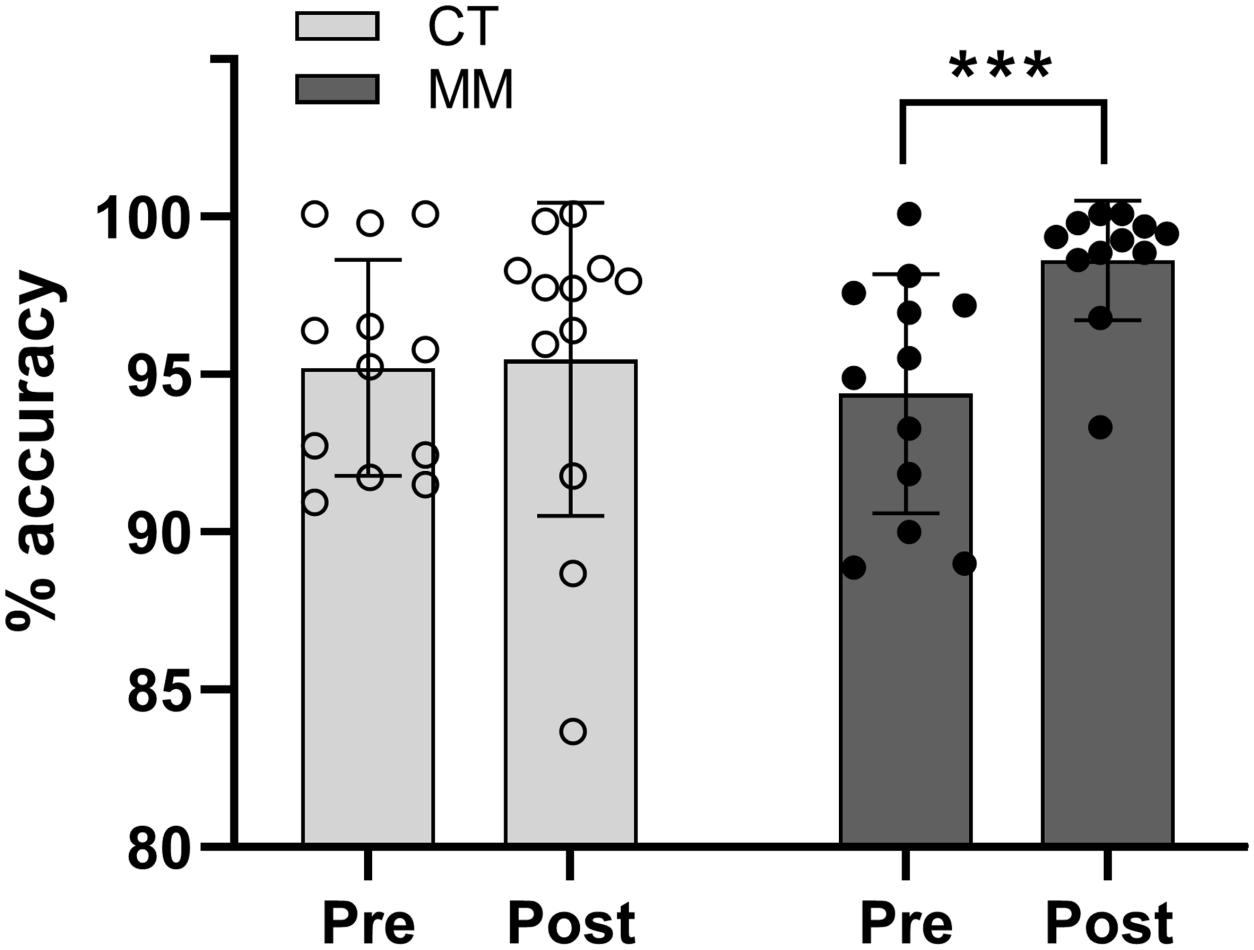
Mean accuracy (%) for the trained hand motor task involving 20 trials of circle-tracing using the dominant hand on Day 1. “Pre” represents the mean accuracy of first 5 trials while “Post” represents the mean accuracy of last 5 trials. CT, Control; MM, Mindfulness meditation. ***Denotes significance *p* < 0.001.

**Figure 4:**
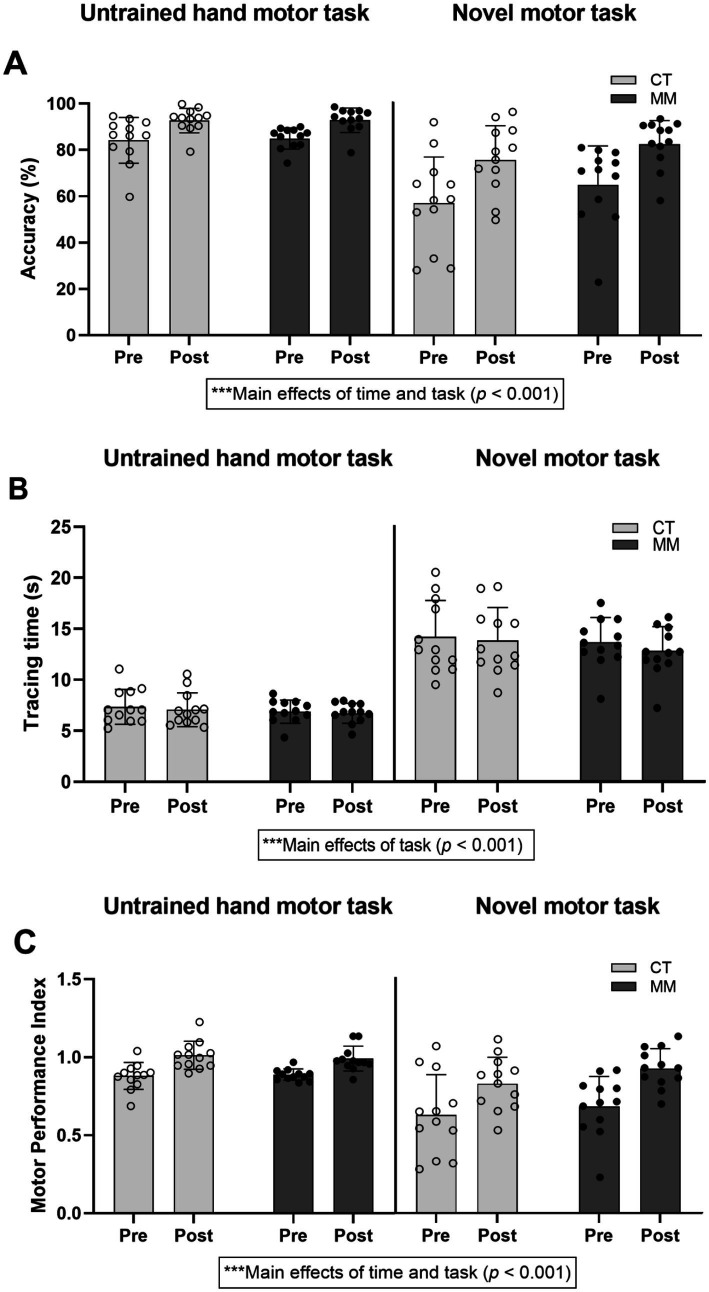
Task accuracy **(A)**, tracing time **(B)**, and motor performance index **(C)** for both the untrained hand motor task involving 30 trials of circle-tracing using the non-dominant hand with time-restriction and novel motor task involving 30 trials of circle-tracing using the non-dominant hand with inverted screen display and time-restriction. “Pre” represents the mean performance of first 5 trials while “Post” represents the mean performance of last 5 trials. CT, control; MM, mindfulness meditation.

For the % accuracy of trained hand motor task, there was a significant main effect of time (*F*_1,2_ = 8.001, *p* = 0.010) with group-time interaction effect (*F*_1,22_ = 6.176, *p* = 0.021). The main effect of group was not significant (*F*_1,22_ = 0.812, *p* = 0.377). For pairwise Pre to Post comparisons, the motor performance was found to be unaltered in the CT group (Pre: *M* = 95.2, 95% CI [93.0, 97.4]; Post: *M* = 95.5, 95% CI [93.3, 97.6], *p* = 0.835). On the contrary, a learning effect, reflected as an improvement in tracing accuracy, was observed in the MM group (Pre: *M* = 94.4, 95% CI [92.2, 96.5]; Post: *M* = 98.6, 95% CI [96.4, 100.8], *p* < 0.001) (Figure 3). Correspondingly, the magnitude of accuracy improvement in the MM group was found to be greater than that of CT group (CT: *M* = +0.3%, *SD* = 4.4; MM: *M* = +4.2%, *SD* = 3.3; *p* = 0.021) (Figure 4).

For the % accuracy of untrained hand and novel motor task, there were significant main effects of time (*F*_1,66_ = 42.400, *p* < 0.001), and task (*F*_1,66_ = 87.124, *p* < 0.001) (Figure 4A). The main effect of group was not significant (*F*_1,22_ = 0.825, *p* = 0.373). None of the two-way or three-way interactions reached significance (all *p* > 0.05) except for the time-task interaction effect (*F*_1,66_ = 5.921, *p* = 0.018). Estimated marginal means indicated that % accuracy increased from pre (*M* = 72.8, 95% CI [68.7, 76.9]) to post (*M* = 85.8, 95% CI [81.7, 89.9]), and were higher for the untrained hand (*M* = 88.7, 95% CI [84.6, 92.8]) than the novel task (*M* = 69.9, 95% CI [65.8, 74.0]). Pairwise comparisons confirmed both differences were significant (both *p* < 0.001, Bonferroni-adjusted).

For the tracing times of untrained hand and novel motor task, there was only significant main effect of task (*F*_1,66_ = 821.139, *p* < 0.001) (Figure 4B). There were no main effects of time (*F*_1,66_ = 3.145, *p* = 0.081) and group (*F*_1,22_ = 0.480, *p* = 0.496). None of the two-way or three-way interactions reached significance (all *p* > 0.05). Estimated marginal means indicated that the tracing times were lower for the untrained hand (*M* = 7.0 s, 95% CI [6.1, 7.9]) than the novel task (*M* = 13.6 s, 95% CI [12.7, 14.5]) where pairwise comparison confirmed the difference was significant (*p* < 0.001, Bonferroni-adjusted).

For MPI scores, there were significant main effects of time (*F*_1,66_ = 41.168, *p* < 0.001), and task (*F*_1,66_ = 45.964, *p* < 0.001) (Figure 4C). The main effect of group was not significant (*F*_1,22_ = 0.825, *p* = 0.373). None of the two-way or three-way interactions reached significance (all *p* > 0.05). Estimated marginal means indicated that MPI scores increased from pre (*M* = 0.77, 95% CI [0.72, 0.82]) to post (*M* = 0.94, 95% CI [0.89, 0.99]), and were higher for the untrained hand (*M* = 0.94, 95% CI [0.90, 0.99]) than the novel task (*M* = 0.77, 95% CI [0.72, 0.81]). Pairwise comparisons confirmed both differences were significant (both *p* < 0.001, Bonferroni-adjusted). Group means did not differ significantly (MM: *M* = 0.84, 95% CI [0.78, 0.89]; CT: *M* = 0.87, 95% CI [0.82, 0.93]; *p* = 0.373). Collectively, the results suggest that the enhanced motor learning performance (reflected as higher MPI scores) from Pre to Post in both groups was driven by an improvement in % accuracy and not tracing times.

### 3.5 Parametric go/no-go test

**Figure 5:**
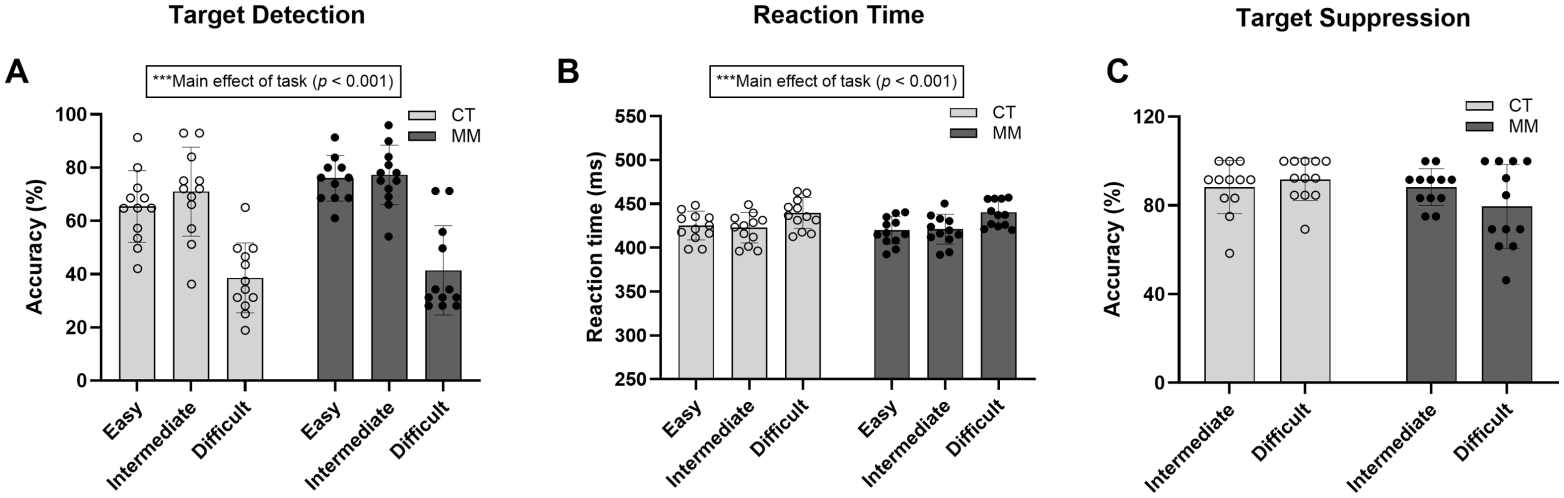
Cognitive test performances for parametric Go/No-Go (PGNG) test encompassing **(A)** Target detection – assessed by response accuracy to correct target trials, **(B)** Reaction time to stimuli, and **(C)** Target suppression – assessed by response accuracy to correct inhibit trials. Similar outcome measures were collated across three difficulty levels of PGNG test – Easy, Intermediate, and Difficult, except there is no element of target suppression in the easy level. CT, Control; MM, Mindfulness meditation.

For target detection, there was significant main effect relating to task difficulty (*F*_2,42.9_ = 49.712, *p* < 0.001) with no group effect observed (*F*_1,21.4_ = 3.380, *p* = 0.081). Both MM and CT groups showed comparable accuracy in “Easy,” “Intermediate” and “Difficult” level of the PGNG test (Figure 5A). Likewise, for reaction time, there was significant main effect of task difficulty (*F*_2,44_ = 25.344, *p* < 0.001) with no group effect observed (*F*_1,22_ = 0.124, *p* = 0.728). Across all difficulty levels, the mean reaction times were comparable between both groups (Figure 5B). For target suppression, no significant task difficulty or group effects (*p* > 0.05) were observed (Figure 5C) (note: there is no element of target suppression in the “Easy” level).

## 4 Discussion

The present study investigated the effect of a single session of guided MM preceding motor learning tasks on anxiety levels and the subsequent learning effect. Our findings suggest that a single session of guided MM can transiently lower HR and feelings of anxiety, as assessed from VAS-A scores. We found that performing guided MM as a priming strategy facilitated the learning of simple motor tasks in healthy young adults.

A state of anxiety impedes motor learning as it is accompanied by the stiffening of the body which results in an increase in jerky and rigid movements (Pijpers et al., 2003). Moreover, high levels of anxiety can disrupt the motor learning process due to disproportionate amount of attention, behaviours, and cognitive resources devoted to unwanted thoughts and feelings (Hoge et al., 2013). Thus, an important mechanism of action in mindfulness-based interventions is to offset the body’s stress response and enhance the ability to intentionally deploy one’s attention, allowing for more flexible cognitive and behavioural responses (Sipe and Eisendrath, 2012). We found that during the brief session of guided MM, the participants’ HR was lowered with improved VAS-A scores. Slow and deep breathing in MM can alleviate bodily symptoms of distress and shift the body into a relaxed state by balancing sympathetic and parasympathetic responses (Kabat-Zinn, 2003). The practice of meditation is typically known to reduce autonomic parameters including heart rate, respiratory rate, blood pressure, and induce a physiologically quiescent state (Khalsa et al., 2015). The observed reduction in HR in our study reflects a transient calming effect brought about by the guided MM session.

However, the reduction in HR was transient and this relaxation state did not persist as the starting mean HR were found to be similar before the commencement of the motor learning tasks. Nonetheless, the reframing of the physiological state before the motor learning tasks could have been important in modulating the baseline attentional state (Sumantry and Stewart, 2021) and reducing the anxiety levels (Strohmaier et al., 2021) of participants. The VAS-A scores were shown to be improved after the guided MM session while STAI-S scores remained comparable. While healthy individuals do experience anxiety during early motor learning (Cabral et al., 2024), their baseline anxiety levels are expected to be low and thus, the benefit may not be as significant as compared to those with higher levels of anxiety. The benefits of MM could be amplified for patients such as stroke survivors with anticipatory anxiety during motor skill learning (Chan et al., 2012; Kou et al., 2024). Furthermore, the use of category rating scales in STAI-S, as opposed to the analogue scale in VAS-A, could have prompted participants to avoid choosing the extreme categories on the scale, which hinders discrimination on graded levels of anxiety.

For the translation of benefits to motor learning, we found that compared to CT, participants in the MM group showed significant learning effects in the simple motor task (circle tracing using motor-competent dominant arm). The benefit to simple motor learning tasks could be attributed to the lowered anxiety after brief MM. It has been found that high anxiety leads to narrowing of visual attention scope (Najmi et al., 2012), reduces attentional resources and impairs gross motor skill learning and performance (Hutchinson and Cotten, 1973; Mullen et al., 2005; Sporn et al., 2020). Furthermore, individuals with anxiety often exhibit impairment of cognitive flexibility and control (Du et al., 2022). Therefore, a lower anxiety level could enhance attentional regulation and cognitive control to support motor learning processes. While the benefit of MM for simpler motor learning is compelling, a similar benefit was not observed for the untrained hand task and novel motor task. Untrained and novel tasks may introduce additional challenges, such as unfamiliarity with non-dominant limb motor control or the simultaneous need for conflict resolution and precise hand-eye coordination, which necessitate more substantial adaptations (e.g., improvement in complex cognitive processing) beyond anxiety reduction to achieve measurable benefits. Moreover, the brief MM session did not enhance performance in PGNG test, indicating unaltered sustained attention, processing speed, or inhibitory control.

The lack of benefits could be a result of the brief duration of the MM session conducted for participants naïve to meditation. MM has been regarded to be a technique that can be improved with practice (Bishop et al., 2004). Attention is likely to benefit most from regular practice as it requires effortful control during the initial sessions but becomes increasingly effortless with experience (Tang and Posner, 2009; Malinowski, 2013). Hence, a dose–response relationship is proposed to exist between the frequency of MM practice and sustained attention outcomes (Wimmer et al., 2020). The preservation of sustained attention may manifest with long-term MM practice. Likewise, the guided MM session lasting only 10 min could be inadequate to improve higher-order cognitive functions such as inhibitory control and processing speeds, especially in novice meditators. Thus, we postulate that trained meditators engaged in regular and/or extended MM sessions may observe better benefits for attentional control to facilitate novel or complex motor learning, and this warrants further investigations.

Some limitations were present in this study. Firstly, it is important to note that the present study did not employ an active control arm which accounts for non-specific effects (Davidson and Kaszniak, 2015) including those arising from differential demand characteristics, expectancy or performance bias, and physiological relaxation. Future studies should compare MM intervention to an active control arm such as an audio listening task sharing similar basic elements and stimulus modality (Chan et al., 2020). Secondly, the varying sports backgrounds and motor competence of participants could have influenced their motor learning and memory retention. To ensure that the motor learning task was equally challenging for participants to learn, ambidextrous individuals were excluded from this study and hand dominance was objectively measured using the Edinburgh Handedness Inventory. Nonetheless, some participants were noted to have engaged in hobbies and/or physical activities requiring fine motor skills and hand-eye coordination (e.g., hockey). Hence, the differing motor skill competence of the participants could have influenced their performance in the motor learning tasks. Finally, the absence of participant blinding could have contributed to potential performance bias despite participants being naïve to the use of MM prior to the study.

Due to the nature of our study design (where the guided MM session precedes motor learning), the PGNG cognitive test could only be performed after the completion of the motor learning tasks. Cognitive assessment was conducted to elucidate any cognitive changes resulting from the brief MM session, given that the participants underwent the same learning tasks. However, the motor learning tasks could have led to differing alterations in attentional state and mental strain in participants prior to the commencement of PGNG test, making it challenging to detect differences, if any. Future studies should explore the use of electroencephalogram (EEG) during the motor learning tasks where real-time recordings of different EEG wave forms can be used to monitor the levels of attention and arousal (Wang et al., 2015; Han et al., 2024).

## 5 Conclusion

In this study, a single session of guided MM reduced anxiety and facilitated the learning of simple motor tasks. However, the effect of a brief MM session on novel or complex motor skill acquisition is still unclear. Nonetheless, our study demonstrated the potential of using MM as a cognitive strategy to support the process of motor learning. Therefore, brief MM may be used as a priming strategy for healthy young adults prior to the learning of motor tasks such as the operating of new machinery in occupational setting, or the acquisition of new sports-related movement. The simplicity and high accessibility of MM support its potential as a low-cost effective tool to facilitate motor skill learning. Future studies may explore the effect of extended MM sessions and/or regular MM practice on novel or more complex motor learning as trained meditators are likely to reap greater benefits on attentional control.

## Data Availability

The datasets presented in this study can be found in online repositories. The names of the repository/repositories and accession number(s) can be found at: https://osf.io/fcgk3/overview?view_only=b6516320aa8544d8b406ff117f6d5608.

## Footnotes

1 https://www.randomlists.com/team-generator.

2 see reference video: https://www.youtube.com/watch?v=zK2TXgGu-GQ.
