# Supplementary material for: Guided mindfulness meditation as a priming strategy for reducing anxiety and facilitating motor skill learning
Source: Front Psychol. 2026 Apr 16;17:1697956. doi: 10.3389/fpsyg.2026.1697956 (PMC13128350; doi:10.3389/fpsyg.2026.1697956)
Supplement: Supplementary file 1 [file Table_1.DOCX]

| Supplementary Table S1: Sensitivity Analysis Table | | | |  |
| --- | --- | --- | --- | --- |
| **% change in Accuracy from Baseline** | **% Time taken (Fixed)** | **% Accuracy** | **MPI** | **% change from previous value** |
| 0.0 | 75.0 | 60.0 | 0.80 | N.A. |
| 5.0 | 75.0 | 63.0 | 0.84 | 5.0 |
| 10.0 | 75.0 | 66.0 | 0.88 | 4.8 |
| 15.0 | 75.0 | 69.0 | 0.92 | 4.5 |
| 20.0 | 75.0 | 72.0 | 0.96 | 4.3 |
| 25.0 | 75.0 | 75.0 | 1.00 | 4.2 |
| 30.0 | 75.0 | 78.0 | 1.04 | 4.0 |
| 35.0 | 75.0 | 81.0 | 1.08 | 3.8 |
| 40.0 | 75.0 | 84.0 | 1.12 | 3.7 |
| 45.0 | 75.0 | 87.0 | 1.16 | 3.6 |
| 50.0 | 75.0 | 90.0 | 1.20 | 3.4 |
|  |  | ****Capped at 100%*** |  |  |
|  |  |  |  |  |
| **% change in Time from Baseline** | **% Time taken** | **% Accuracy (Fixed)** | **MPI** | **% change from previous value** |
| 0.0 | 60.0 | 75.0 | 1.25 | N.A. |
| 5.0 | 63.0 | 75.0 | 1.19 | -4.8 |
| 10.0 | 66.0 | 75.0 | 1.14 | -4.5 |
| 15.0 | 69.0 | 75.0 | 1.09 | -4.3 |
| 20.0 | 72.0 | 75.0 | 1.04 | -4.2 |
| 25.0 | 75.0 | 75.0 | 1.00 | -4.0 |
| 30.0 | 78.0 | 75.0 | 0.96 | -3.8 |
| 35.0 | 81.0 | 75.0 | 0.93 | -3.7 |
| 40.0 | 84.0 | 75.0 | 0.89 | -3.6 |
| 45.0 | 87.0 | 75.0 | 0.86 | -3.4 |
| 50.0 | 90.0 | 75.0 | 0.83 | -3.3 |
|  | ****Capped at 100%*** |  |  |  |
