# Supplementary material for: Guided mindfulness meditation as a priming strategy for reducing anxiety and facilitating motor skill learning
Source: Front Psychol. 2026 Apr 16;17:1697956. doi: 10.3389/fpsyg.2026.1697956 (PMC13128350; doi:10.3389/fpsyg.2026.1697956)
Supplement: Supplementary file 2 [file Table_2.DOCX]

Supplementary Table S2: Time Scaling Effect Assessment

| **Allocated time for tracing (s)** | **Reference records (n)** | **Average % improvement attained below allocated time** | **Standard deviation** |
| --- | --- | --- | --- |
| 5 | 2 | 36.5 | 30.9 |
| 6 | 10 | 38.5 | 13.7 |
| 7 | 13 | 43.5 | 14.6 |
| 8 | 10 | 48.6 | 20.5 |
| 9 | 6 | 22.9 | 7.0 |
| 10 | 4 | 41.0 | 25.2 |
| 11 | 1 | 51.2 | NA |
| 12 | 4 | 44.5 | 12.7 |
| 13 | 7 | 54.1 | 17.3 |
| 14 | 6 | 38.6 | 24.3 |
| 15 | 6 | 31.7 | 9.8 |
| 16 | 3 | 48.3 | 7.7 |
| 17 | 1 | 46.9 | NA |
| 18 | 4 | 35.2 | 18.1 |
| 19 | 1 | 42.2 | NA |
| 21 | 2 | 22.9 | 12.8 |
